# Advanced Behavioral Analyses Show that the Presence of Food Causes Subtle Changes in *C. elegans* Movement

**DOI:** 10.3389/fnbeh.2016.00060

**Published:** 2016-03-31

**Authors:** Nicholas B. Angstman, Hans-Georg Frank, Christoph Schmitz

**Affiliations:** Department of Neuroanatomy, Ludwig-Maximilians University of MunichMunich, Germany

**Keywords:** *C. elegans*, *E. coli*, food, locomotion, tracking

## Abstract

As a widely used and studied model organism, *Caenorhabditis elegans* worms offer the ability to investigate implications of behavioral change. Although, investigation of *C. elegans* behavioral traits has been shown, analysis is often narrowed down to measurements based off a single point, and thus cannot pick up on subtle behavioral and morphological changes. In the present study videos were captured of four different *C. elegans* strains grown in liquid cultures and transferred to NGM-agar plates with an *E. coli* lawn or with no lawn. Using an advanced software, WormLab, the full skeleton and outline of worms were tracked to determine whether the presence of food affects behavioral traits. In all seven investigated parameters, statistically significant differences were found in worm behavior between those moving on NGM-agar plates with an *E. coli* lawn and NGM-agar plates with no lawn. Furthermore, multiple test groups showed differences in interaction between variables as the parameters that significantly correlated statistically with speed of locomotion varied. In the present study, we demonstrate the validity of a model to analyze *C. elegans* behavior beyond simple speed of locomotion. The need to account for a nested design while performing statistical analyses in similar studies is also demonstrated. With extended analyses, *C. elegans* behavioral change can be investigated with greater sensitivity, which could have wide utility in fields such as, but not limited to, toxicology, drug discovery, and RNAi screening.

## Introduction

As a highly-used model organism, *Caenorhabditis elegans* (*C. elegans*) worms offer a wide range of possibilities for scientific research. With advantages including, but not limited to, easy maintenance and handling, forward and reverse genetic manipulability (Jorgensen and Mango, 2002), and a fully mapped nervous system connectome of 302 neurons (White et al., 1986), *C. elegans* offer an alternative to larger rodent model organisms. Furthermore, *C. elegans* can be cultured in both liquid media and on agar plates (Sulston and Brenner, 1974; Angstman et al., 2015) and fed with concentrated *Escherichia coli* (*E. coli*).

Behavioral analysis of *C. elegans* is generally performed on NGM-agar plates in a quasi 2D model under a dissecting microscope using video capturing software, although assays measuring behavior in liquid have been demonstrated (Hardaway et al., 2014; Restif et al., 2014), as have assays in behavioral arenas (Albrecht and Bargmann, 2011). As NGM-agar plate behavior assays remain the status quo, various tracking software is available to generate a readout on such behavior (summarized in Husson et al., 2012). Features of such software range from single worm tracking to multi-worm tracking, and from single point (centroid) tracking to full worm outline tracking (Table 1). Although, readouts using methods are often limited to speed of locomotion, more complicated behavioral phenotypes such as omega bending and reversals have been defined and detected (Huang et al., 2006). Some authors have attempted to identify as many as 702 features of worm movement by splitting certain features into sub-features (for example breaking up tail motion direction into forward, paused, and negative tail motion direction; Yemini et al., 2013).

**Table 1:** **Summary of ***C. elegans*** tracking software features**.

**Tracker**	**Nemo^a^**	**The parallel worm Tracker^b^**	**OptoTracker^c^**	**Multimodal illumination and tracking system^d^**	**CoLBeRT^e^**	**The multi worm tracker^f^**	**Optomechanical system for virtual environments^g^**	**Worm Tracker 2.0^h^**	**WormLab^i^**
Single/Multiple worms	Single	Multiple	Multiple	Single	Single	Multiple	Single	Single	Multiple
Tracking capability	Skeleton and outline	Mid-point	Mid-point	Skeleton and outline	Skeleton and outline	Skeleton and outline	Bright spot	Skeleton and outline	Skeleton and outline

a Tsibidis and Tavernarakis, 2007;

b Ramot et al., 2008;

c Ramot et al., 2008;

d Stirman et al., 2011;

e Leifer et al., 2011;

f Swierczek et al., 2011;

g Faumont et al., 2011;

h Geng et al., 2004;

i *Roussel et al., 2014*.

As *C. elegans* are often raised on NGM-agar plates with an *E. coli* lawn, behavioral assays can be performed in the presence of food or on bare NGM-agar plates. The presence of food has previously been shown to decrease *C. elegans* speed of locomotion (Ramot et al., 2008). Thus, the effect of an *E. coli* lawn in a behavioral assay is of relevance to, at the very least, investigations of feeding related genes (see, e.g., de Bono and Bargmann, 1998).

In the present study we demonstrate that statistically significant behavioral differences beyond speed of locomotion can be observed when investigating four different strains of *C. elegans* on either NGM-agar plates with an *E. coli* lawn or with no lawn. Furthermore, we show that such a change in environment also affects the relationship of speed with other behavioral traits—that is behavioral change can be observed even in the lack of an observed change in speed of locomotion. Specifically we tested the hypothesis that various behavioral traits can be measured to show the effect of the presence of *E. coli* in the moving properties of *C. elegans*.

## Materials and methods

### Nematodes

Wild type (N2, Bristol), DA508 *npr-1(n1353)*, DA609 *npr-1(ad609)*, and CX4148 *npr-1(ky13) C. elegans*, as well as OP50 *E. coli* were obtained from the Caenorhabditis Genetics Center (Minneapolis, MN, USA), which is funded by NIH Office of Research Infrastructure Programs (P40 OD010440). Mutant *npr-1* strains of *C. elegans* were selected based on previous findings that such strains are hyperactive on plates containing food as compared to wild type worms (de Bono and Bargmann, 1998). From a stock liquid culture, synchronous young adult worms were produced via sodium hypochlorite treatment and sucrose cleaning as described in detail in Angstman et al. (2015). Worms were raised in liquid cultures at 24°C in an incubated shaker (NB 205V, N Biotek, Bucheon, South Korea).

### Assay

From a tube of worms in S-Medium, 10 μl containing 15–30 worms were pipetted on to a modified membrane filter-vacuum filtration system (Angstman et al., 2015). Following removal of liquid, membrane filters were flipped and placed on to a 6 cm NGM-agar plate (see Supplemental Video 2 in Angstman et al., 2015). Plates contained either an *E. coli* lawn (created by spreading over the agar surface and allowing to grow overnight) or no lawn (control).

**Figure 1:**
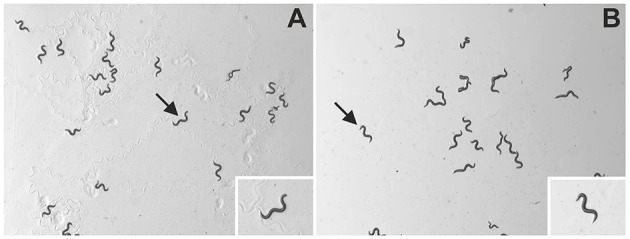
**Representative images of ***C. elegans*** as analyzed in the present study**. The panels show unprocessed frames of videos of wild type (N2) *C. elegans* on NGM-agar plates with an *E. coli* lawn **(A)** or with no lawn **(B)**. The insets show individual worms (indicated by arrows) at higher magnification. The slightly uneven illumination of the panels stems from the fact that in the experimental setup of the present study, the field-of-view was placed offset from the middle of the agar plates. However, this had no impact on unequivocal identification and tracking of the worms on the plates (evaluation data not shown).

Plates were immediately placed under a dissecting microscope (MZ75; Leica, Wetzlar, Germany; equipped with 1.0X PlanApo objective) with an LCD light source with a color temperature of 2800 K (KL 1500; Schott, Mainz, Germany). Using the video capture function of the software, WormLab (Version 3.0.0, MBF Bioscience, Williston, VT, USA), 60 s long videos with a resolution of 1280 × 960 pixels were taken at 15 frames/s using a mono digital camera (Grasshopper 2, Point Grey Research, Richmond, BC, Canada). Using a horizontal mm ruler and the measure function of WormLab, videos were determined to have a scale of 8.37 μm/pixel, which enabled precise and accurate investigation of the parameters described in the next section. Accordingly, the field-of-view of the camera was 10.7 by 8.0 mm and, thus, 3% of the base area of the agar plates (Figure 1 and Supplementary Videos S1, S2).

At the beginning of each video plates were adjusted so that all worms were within the field-of-view of the camera. All worms that were tracked for >75 of the first 150 frames were included in the final analysis, with track lengths of up to 900 frames possible. Tracks that started after the first 150 frames were excluded from the analysis because they could have represented worms that initially moved out of but then came back into the field-of-view of the camera. These parameters avoided the possibility of double counting individual worms.

Captured videos were tracked with WormLab using settings outlined in Supplementary Table 1. Results from each video including Position–Midpoint (x, y), Bending Angle-Mid-Point, Wavelength, Omega Bend, and Reversal were exported and processed using Microsoft Excel 2010 (Microsoft, Redmond, WA, USA).

### Investigated parameters

#### Speed of locomotion

Speed of worms was calculated from Position to Midpoint (x, y) data as in our earlier study (Angstman et al., 2015). Direction was not taken into account. Using coordinates, speed was calculated for each second as the distance traveled over 15 frames. Single point values of >500 μm/s were considered outliers and removed from the data set. Each qualifying worm was represented with one value for average speed determined by the arithmetic mean of the values calculated each second.

#### Bending angle

**Figure 2:**
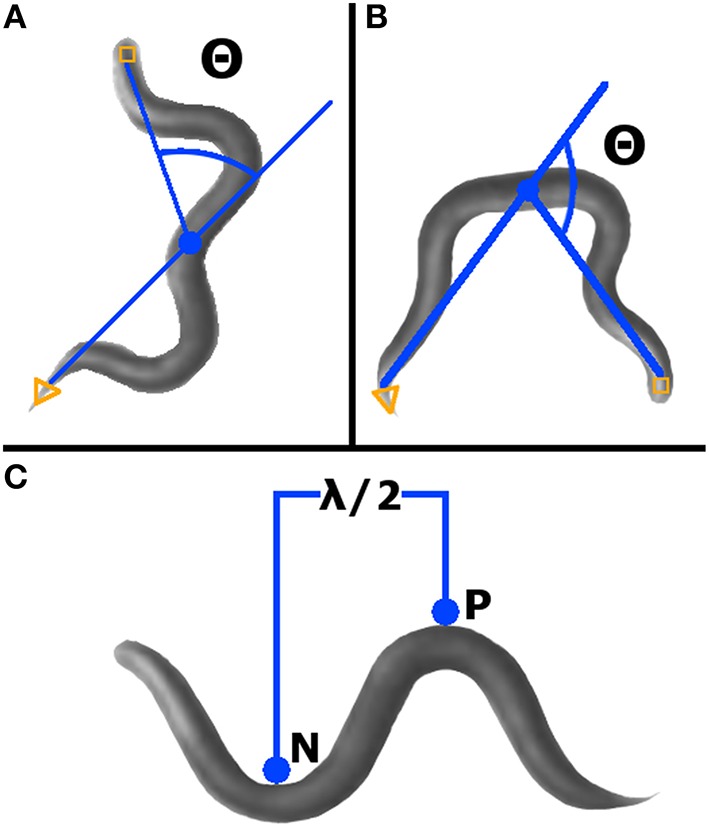
**WormLab advanced analyses. (A)** Bending angle (Θ) was measured as the supplement of the angle between the head, mid-point, and tail. Worms with head, mid-point, and tail in line have an angle of zero. **(B)** Omega bending is indicated when the angle (Θ) created between the head, mid-point, and tail is < 90° (i.e., the bending angle is >90°). **(C)** Wavelength was measured as two times the distance between the positive (P) and negative (N) stationary points.

The bending angle is defined as the angle between the midpoint-head and midpoint-tail segments, with a straight worm set as zero degrees (Figure 2A). For a given worm, a bending angle was measured for each frame tracked. In the final readout of average bending angle for a given worm, the absolute value of the angle measured in each frame was averaged. Standard deviation of all absolute value bending angles was calculated for each qualifying worm.

#### Omega bending

As defined by WormLab, omega bending occurs when the bending angle exceeds 90 degrees and continues until the bending angle goes below 90° (Figure 2B). Omega bending was counted only when a minimum duration of 10 frames was reached. For each worm meeting qualifying requirements, omega bending was expressed as a percentage of frames tracked.

#### Wavelength

This parameter is defined as the two times the measurement between the positive and the negative inflection points (Figure 2C). Wavelength was calculated for each worm in each frame tracked, except when at zero or only one inflection point was found. For qualifying worms, average wavelength was expressed as the average of all calculated wavelengths (i.e., from each frame tracked).

#### Reversal frequency

This parameter was calculated based on smoothed speed provided by WormLab, a smoothed moving average speed taken over a 20 frame span (based on Cleveland and Devlin, 1988). In order to remove bias from possible incorrect head/tail recognition, smoothed speed was inversed if over half of the frames tracked were measured as negative speed values. Reversal was then counted only in stretches of negative smoothed speed ≥10 frames. Final results were reported for each qualifying worm as a percentage of reversals per frame tracked.

### Statistical analysis

In the final analyses of NGM-agar plates with an *E. coli* lawn, 172 N2 worms on 10 different plates, 189 *npr-1(n1353)* worms on six different plates, 132 *npr-1(ad609)* worms on six different plates and 129 *npr-1(ky13)* worms on six different plates were counted. Analyses of NGM-agar plates with no lawn included 173 N2 worms on 10 different plates, 159 *npr-1(n1353)* worms on six different plates, 139 *npr-1(ad609)* worms on six different plates, and 167 *npr-1(ky13)* worms on six different plates.

Due to the fact that several worms were analyzed per plate, a nested ANOVA design was used to determine the significance of (i) lawn type (i.e., plates with an *E. coli* lawn vs. plates with no lawn) and (ii) whether the nested nature of this assay contributed to observed differences (c.f. Aarts et al., 2014). In the nested ANOVA, the various investigated parameters were used as the dependent variable, while lawn type was a fixed factor, and plate number was a random factor.

To investigate correlation between analyses, the runs test (c.f. Wald and Wolfowitz, 1940) was used to determine if relationships departed from linearity. If a statistically significant departure was not found, linear regression was used to compare analyses. If departure from linearity was statistically significant, non-parametric correlation (Spearman) was used.

Nested ANOVA was performed in SPSS (Version 23 for Windows; IBM, Armonk, NY, USA), while all other statistical analyses were performed in GraphPad Prism (version 5.04 for Windows; GraphPad Software, San Diego, CA, USA).

A *p*-value of 0.05 was used as the criterion for statistical significance in all analyses.

## Results

### Lawn vs. no lawn

The results of comparisons between worms on NGM-agar plates with an *E. coli* and worms on NGM-agar plates with no lawn are summarized in detail in Table 2. Lawn type was found to result in a statistically significant difference in 25/28 analyses across the four strains of *C. elegans*.

**Table 2:** **Summary of analyses and comparison between ***C. elegans*** on plates with ***E. coli*** and no lawn**.

**Analysis**	**Strain**	**Lawn Type**											
		***E. coli***		**No Lawn**		**Lawn Type**				**Plate (Lawn Type)**			
		***Mean***	***SD***	***Mean***	***SD***	***df1***	***df2***	***F***	***p***	***df1***	***df2***	***F***	***p***
Average speed (μm/s)	N2	128.6	41.6	146.9	55.3	1	19.324	4.4	**0.049**	18	325	2.394	**0.001**
	*npr-1(n1353)*	110.8	39.6	130.4	49.0	1	10.226	5.2	**0.045**	10	336	3.679	**0.000**
	*npr-1(ad609)*	119.2	53.5	83.4	46.0	1	10.467	17.5	**0.002**	10	259	1.814	0.058
	*npr-1(ky13)*	165.9	182.4	182.4	62.7	1	10.551	2.5	0.140	10	298	2.129	**0.022**
Average Angle (°)	N2	34.3	14.2	40.0	17.1	1	19.387	6.3	**0.022**	18	325	2.287	**0.002**
	*npr-1(n1353)*	25.0	12.7	44.8	20.5	1	10.248	38.0	**0.000**	10	336	3.359	**0.000**
	*npr-1(ad609)*	28.2	12.1	48.9	24.3	1	11.108	98.6	**0.000**	10	259	0.776	0.652
	*npr-1(ky13)*	26.7	11.1	36.1	17.7	1	12.138	42.5	**0.000**	10	298	0.568	0.840
St. Dev. Angle (°)	N2	24.3	9.5	29.1	12.1	1	20.047	11.3	**0.003**	18	325	1.562	0.068
	*npr-1(n1353)*	17.4	8.7	31.1	13.1	1	10.367	59.5	**0.000**	10	336	2.272	**0.014**
	*npr-1(ad609)*	21.4	10.8	33.0	14.9	1	10.676	47.6	**0.000**	10	259	1.261	0.253
	*npr-1(ky13)*	18.1	6.6	25.6	12.7	1	11.453	36.9	**0.000**	10	298	0.824	0.606
Average wavelength (μm)	N2	383.0	42.7	347.5	43.9	1	19.931	36.9	**0.000**	18	325	1.653	**0.046**
	*npr-1(n1353)*	363.2	52.0	330.0	53.3	1	10.398	15.7	**0.002**	10	336	2.097	**0.024**
	*npr-1(ad609)*	346.1	70.2	318.2	67.3	1	10.580	6.3	**0.030**	10	259	1.465	0.153
	*npr-1(ky13)*	411.3	45.2	371.8	60.9	1	10.819	29.0	**0.000**	10	298	1.441	0.161
St. Dev. wavelength (μm)	N2	103.3	31.3	91.8	34.3	1	19.533	4.6	**0.045**	18	325	2.073	**0.007**
	*npr-1(n1353)*	103.4	33.4	90.9	30.8	1	10.419	6.8	**0.025**	10	336	1.996	**0.033**
	*npr-1(ad609)*	85.0	31.6	99.3	37.8	1	10.451	6.6	**0.027**	10	259	1.880	**0.048**
	*npr-1(ky13)*	93.1	31.9	86.1	35.4	1	10.803	3.1	0.108	10	298	1.468	0.150
Omega bending percent	N2	2.25	7.69	3.99	8.82	1	20.413	3.7	0.069	18	325	1.331	0.166
	*npr-1(n1353)*	0.82	4.08	7.31	11.61	1	10.440	27.1	**0.000**	10	336	1.900	**0.044**
	*npr-1(ad609)*	1.09	3.48	8.89	15.76	1	17.234	172.5	**0.000**	10	259	0.133	0.999
	*npr-1(ky13)*	0.08	0.75	3.00	9.12	1	11.394	9.0	**0.012**	10	298	0.857	0.574
Reversal percent	N2	2.90	6.59	5.88	10.44	1	21.214	8.7	**0.008**	18	325	1.008	0.449
	*npr-1(n1353)*	3.19	8.01	14.57	14.77	1	10.259	28.3	**0.000**	10	336	3.215	**0.001**
	*npr-1(ad609)*	8.22	12.22	21.63	13.23	1	10.566	49.7	**0.000**	10	259	1.501	0.139
	*npr-1(ky13)*	2.24	6.77	4.68	10.41	1	11.830	8.4	**0.014**	10	298	0.659	0.762

*P-values smaller than 0.05 are given boldface*.

**Figure 3:**
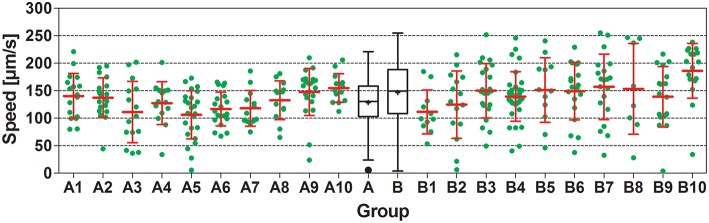
**Speed of locomotion of N2 (wild type) ***C. elegans*** on NGM agar plates with an ***E. coli*** lawn and on plates with no lawn**. Groups A1–A10 and B1–B10 show individual data (green dots) and mean ± standard deviation (red lines) of speed of locomotion of N2 worms on 10 plates with an *E. coli* lawn (Groups A1–A10) and on 10 plates with no lawn (Groups B1–B10). Groups A and B show Tukey boxplots of the speed of locomotion of all worms on plates with an *E. coli* lawn (Group A) and of all worms on plates with no lawn (Group B). Nested ANOVA showed a statistically significant difference between worms on plates with an *E. coli* lawn and worms on plates with no lawn (*p* = 0.049) as well as a statistically significant effect among plates (*p* = 0.001; see also Table 2).

In all experiments carried out, worms showed substantial interindividual variation in all investigated parameters. For example, speed of locomotion of N2 worms on plates with an *E. coli lawn* varied between 5.5 and 221 μm/s, and on plates with no lawn between 4.0 and 255 μm/s (Figure 3). Besides this, mean values of all investigated parameters showed considerable inter-plate variability. For example, mean speed of locomotion of N2 worms on plates with an *E. coli* lawn varied between 106 and 155 μm/s among plates, and on plates with no lawn between 112 and 186 μm/s among plates (Figure 3).

### Plate effect

The nested factor—accounting for the fact that multiple specimen can be grouped by plate—accounted for statistically significant difference in 13/28 analyses (all results of statistical analysis are summarized in Table 2). Both lawn type and the nested factor were statistically significant in 12 cases. Lawn type, but not the nested factor, was statistically significant in 13 cases, while the nested factor, but not lawn type, was statistically significant in only one case. In only two cases was neither lawn type nor the nested factor statistically significant.

### Correlation of speed with various behavioral analyses

The results of these analyses are summarized in Table 3 for *C. elegans* on plates with an *E. coli* lawn and in Table 4 for *C. elegans* on plates with no lawn. When comparing average speed to other analyses in worms on an *E. coli* lawn, statistically significant departure from linearity was found in only five cases out of 24 (six correlations across four strains). Departure from linearity was observed in 8/24 cases for worms on plates with no lawn. Linear regression and Spearman non-linear correlation yielded statistical significance in 12 cases for worms on NGM-agar plates with an *E. coli* lawn and 20 cases for worms on NGM-agar plates with no lawn.

**Table 3:** **Summary of correlation between analyses for worms on plates with an ***E. coli*** lawn**.

**Calculation**	**Strain**	**Runs test**			**Linear regression**			**Non-linear correlation**	
		**Points Above line**	**Points Below line**	***p***	***r^2^***	***F***	***p***	***Spearman***	***p***
Average speed vs. average angle	N2	72	100	0.917	0.030	5.277	**0.023**		
	*npr-1(n1353)*	74	115	0.242	0.093	19.11	<**0.001**		
	*npr-1(ad609)*	55	77	0.315	0.01949	2.585	0.110		
	*npr-1(ky13)*	56	73	0.508	0.232	38.43	<**0.001**		
Average speed vs. St. Dev. angle	N2	76	96	0.810	0.001	0.232	0.631		
	*npr-1(n1353)*	74	115	0.699	0.014	2.697	0.102		
	*npr-1(ad609)*	52	80	0.792	0.002	0.310	0.579		
	*npr-1(ky13)*	62	67	0.766	0.014	1.824	0.179		
Average speed vs. average wavelength	N2	83	89	0.129	0.006	1.042	0.309		
	*npr-1(n1353)*	98	91	0.962	0.108	22.61	<**0.001**		
	*npr-1(ad609)*	67	65	0.397	0.403	87.78	<**0.001**		
	*npr-1(ky13)*	67	62	0.577	0.207	33.06	<**0.001**		
Average speed vs. St. Dev. wavelength	N2	87	85	0.649	0.178	36.93	<**0.001**		
	*npr-1(n1353)*	87	102	0.214	0.108	22.73	<**0.001**		
	*npr-1(ad609)*	60	72	0.301	0.017	2.219	0.139		
	*npr-1(ky13)*	64	65	0.430	0.016	2.048	0.155		
Average speed vs. omega bending Pct	N2	25	147	0.258	0.006	0.989	0.322		
	*npr-1(n1353)*	45	144	<**0.001**				–0.098	0.178
	*npr-1(ad609)*	17	115	0.344	0.008	1.084	0.300		
	*npr-1(ky13)*	22	107	<**0.001**				0.127	0.152
Average speed vs. reversal Pct	N2	54	118	**0.005**				–0.448	<**0.001**
	*npr-1(n1353)*	60	129	<**0.001**				–0.363	<**0.001**
	*npr-1(ad609)*	45	87	0.064	0.202	32.82	<**0.001**		
	*npr-1(ky13)*	32	97	<**0.001**				–0.314	**0.003**

*P-values smaller than 0.05 are given boldface*.

**Table 4:** **Summary of correlation between analyses for worms on plates with no lawn**.

**Calculation**	**Strain**	**Runs test**			**Linear regression**			**Non-linear correlation**	
		**Points above line**	**Points below line**	***p***	***r^2^***	***F***	***p***	**Spearman**	***p***
Average speed vs. average angle	N2	73	100	**0.032**				–0.2428	**0.001**
	*npr-1(n1353)*	71	88	0.680	0.316	72.63	<**0.001**		
	*npr-1(ad609)*	59	80	**0.010**				0.085	0.323
	*npr-1(ky13)*	69	98	0.088	0.164	32.31	<**0.001**		
Average speed vs. St. Dev. angle	N2	74	99	**0.006**				–0.198	**0.009**
	*npr-1(n1353)*	70	89	0.172	0.059	9.913	**0.002**		
	*npr-1(ad609)*	70	69	<**0.001**				0.207	**0.015**
	*npr-1(ky13)*	67	100	0.177	0.076	13.62	<**0.001**		
Average speed vs. average wavelength	N2	88	85	0.501	0.044	7.806	**0.006**		
	*npr-1(n1353)*	83	76	0.873	0.193	37.48	<**0.001**		
	*npr-1(ad609)*	67	72	0.639	0.065	9.456	**0.003**		
	*npr-1(ky13)*	77	90	0.593	0.170	33.80	<**0.001**		
Average speed vs. St. Dev. wavelength	N2	82	91	0.233	0.220	48.34	<**0.001**		
	*npr-1(n1353)*	82	77	0.901	0.178	33.94	<**0.001**		
	*npr-1(ad609)*	67	72	0.887	0.067	9.837	**0.002**		
	*npr-1(ky13)*	79	88	0.900	0.163	32.20	<**0.001**		
Average speed vs. omega bending Pct	N2	44	129	**0.037**				–0.130	0.089
	*npr-1(n1353)*	54	105	0.198	0.060	9.997	**0.002**		
	*npr-1(ad609)*	45	94	0.054	< 0.0001	0.070	0.791		
	*npr-1(ky13)*	30	137	0.153	0.043	7.466	**0.007**		
Average speed vs. reversal Pct	N2	63	110	**0.029**				–0.390	<**0.001**
	*npr-1(n1353)*	69	90	<**0.001**				–0.624	<**0.001**
	*npr-1(ad609)*	68	71	0.307	0.252	46.14	<**0.001**		
	*npr-1(ky13)*	65	102	<**0.001**				–0.436	<**0.001**

*P-values smaller than 0.05 are given boldface*.

## Discussion

### Summary of results

The main results of the present study can be summarized as follows: advanced *C. elegans* behavioral traits, in both wild type and mutant strains, can be used to demonstrate behavioral differences between test groups. Furthermore, such behavioral traits go beyond the standard measure of speed of locomotion and often either do not significantly correlate statistically with speed of locomotion or only correlate with speed of locomotion within certain test groups. Only one analysis, reversal percentage, was found to correlate with average speed of locomotion in all cases. High interindividual variability within analyses was observed, in line with our previously shown data (Angstman et al., 2015), and inter-plate variability was also shown to be a factor in some differences seen in the analyses. This reinforces the need for large sample sizes for behavioral analyses of *C. elegans*, and also shows the importance of using nested analyses when assaying behavior of multiple worms per NGM-agar plate. Using the methods demonstrated in the present study, the ability to accurately measure advanced traits of *C. elegans* behavior offers enhanced functionality in *C. elegans* research.

### Relevance

The comparison of *C. elegans* worms moving on NGM-agar plates with an *E. coli* lawn to worms moving on NGM-agar plates with no lawn represents a relevant biological example for behavioral change in *C. elegans*. This methodology has been used previously using behavioral analysis on N2 worms (Schwarz et al., 2015). The *npr-1* strains used in the present study are described in this context as well. This methodology and these strains were, for example, used to identify genes that play a role in feeding in *C. elegans* (de Bono and Bargmann, 1998; de Bono et al., 2002). The behavioral traits used in the present study were also identified as characteristics of *C. elegans* behavior in the literature (e.g., Buckingham and Sattelle, 2008).

At first glance, it seems that the results of the present study are not in line with certain data reported by de Bono and Bargmann (1998) and de Bono et al. (2002). For example, de Bono and Bargmann (1998) found an increase in mean speed of locomotion of wildtype (N2) *C. elegans* of ~110 μm/s on plates with an *E. coli* lawn to ~300 μm/s on plates with no lawn (+170%; in de Bono et al.'s (2002) study this difference was +225%). This difference is much greater than what was found in the present study (the mean speed of locomotion of N2 *C. elegans* was 128.6 μm/s on plates with an *E. coli* lawn and 146.9 μm/s (+14%) on plates with no lawn; Table 2). However, it is important to note that in the study of de Bono and Bargmann (1998), each average value represented the average speed of locomotion of at least 24 animals investigated on an unknown number of plates during more than 72 min of recording, and in the study of de Bono et al. (2002) each average value represented the average speed of locomotion of at least 25 animals investigated on an unknown number of plates during 4 min of recording. In the present study, the average values of the N2 worms represented the average speed of locomotion during 60 s of recording of 172 worms investigated on 10 plates with an E. coli lawn and 173 worms investigated on 10 plates with no lawn. Unfortunately, de Bono and Bargmann (1998) and de Bono et al. (2002) did not provide essential information about the video recording settings used in their experiments (i.e., the magnification of the objective lens, resolution of the digital camera, scale of the pixels, and size of the field-of-view of the camera). Thus, one cannot determine the relation between the size of the field-of-view of the camera and the size of the circular area in which worms could move during the recording time [this area had a diameter of 2.5 cm in the study of de Bono and Bargmann (1998) and 2.0 cm in the study of de Bono et al. (2002)]. As a result, it remains unknown how worms were handled that moved out of the field-of-view (or moved into the field-of-view, respectively) of the camera particularly during the long recording time of 72 min in the study of de Bono and Bargmann (1998). Because de Bono and Bargmann (1998) and de Bono et al. (2002) did not report standard deviations of speed of movement or potential alterations in speed of movement over time, the question remains open as to whether worms showed a relatively constant speed of movement during the recording times in these studies or different speed of movement at the beginning, middle, and end of the recording times. In this regard, a study by Swierczek et al. (2011) should be kept in mind in which N2 *C. elegans* showed a drop in average speed of locomotion from ~230 μm/s immediately after placing worms on agar plates to ~80 μm/s 10 min later (mean of eight plates). In the present study, videos were taken immediately after worms were placed on agar plates; however, the average speed of locomotion of N2 *C. elegans* on plates with an *E. coli* lawn was only ~55% of the results reported by Swierczek et al. (2011). In summary, in order to ensure reproducibilty of a study analyzing the behavior of *C. elegans* it is essential to provide information about the video recording settings used in the experiments in sufficient detail to enable comparisons of the outcome of different studies.

### Advanced behavioral analysis

In order to offer advanced *C. elegans* behavioral analysis, full worm “skeleton and outline” tracing must be present in order to represent full worm behavior and posture rather than only the simple centroid. Some worm trackers described in the literature offer these capabilities, however most trackers that offer skeleton and outline tracking can track only one worm at a time, while most trackers that can track multiple worms offer only centroid tracking (Table 1). With these capabilities, the advanced behavioral analyses performed in the present study are either, in the latter case, not possible, or in the former case must be done by tracking only one worm at a time. As demonstrated here, large interindividual variability creates the need for a large sample size to reliably compare test groups, making a low-throughput, one-worm-at-a-time solution ineffective due to the time required to generate an adequate sample size. In two systems, Multi Worm Tracker (Swierczek et al., 2011) and WormLab (Roussel et al., 2014), both multi-worm tracking and skeleton and outline tracking are provided together.

In the present study, the video recording time was restricted to 60 s in order to limit worms moving out of or moving back into the field-of-view of the camera. However, it was not possible to decrease the magnification at which the videos were recorded. The latter would have resulted in an increased scale of the pixels and, thus, a decreased number of pixels per worm, preventing precise and accurate advanced behavioral analysis as outlined in the present study. In this regard it should be noted that Swierczek et al. (2011) used a camera with a resolution of 2352 × 1728 pixels and performed video tracking of *C. elegans* at a scale of 24.3 μm/pixel. Accordingly, the field-of-view was 24 cm^2^ in their study and, thus, larger than the base area of the 5 cm NGM-agar plates used by Swierczek et al. (2011). This is substantially different from the settings used in the present study. Swierczek et al. (2011) reported a position jitter of only 1 μm and a speed jitter of only 1 μm/s when analyzing moving *C. elegans* using these settings, which is similar to what was reported for the WormLab software (Roussel et al., 2014). On the other hand, Swierczek et al. (2011) reported that identity of worms was lost upon collision with another worm, which is mitigated using the settings used in the present study (c.f. Supplemental Video 2 in Angstman et al., 2015).

### Measurement of subtle behavioral change

By measuring behavioral change of worms on plates with and without the presence of *E. coli*, it was demonstrated that certain *C. elegans* strains demonstrate substantially increased grouping behavior in the presence of *E. coli* (de Bono et al., 2002). Furthermore, the same study showed reduction in speed in multiple *C. elegans* strains when exposed to food, while also showing that certain deletion mutations affected this phenomenon. In this case, behavioral change was able to shed light on the roles of individual proteins on *C. elegans* behavioral phenomena.

Further analysis of behavioral change of N2 worms in the presence of an *E. coli* lawn vs. no lawn has been demonstrated using tracking of individual worms (Schwarz et al., 2015). It should be noted that this methodology differs from that of the present study in a number of ways: (i) individual worms are used rather than groups of worms on plates, (ii) worms on plates are allowed to habituate for a 30 min period before assaying, while no habituation time is allowed in the present study (in both studies, worms on plates with no lawn are assayed immediately), and (iii) video recording is performed for 15 min, while in the present study video recording consisted of 1 min. In-depth measurement of individual *C. elegans* posture and its change over time, as demonstrated in Schwarz et al. (2015), could be useful, for example, in the understanding of the neurological functions causing various behaviors, but differences in the methodology used between this and the present study demonstrate two differing scopes.

The present study shows that behavioral change, using the example of *E. coli* lawn presence and four different *C. elegans* strains, can go far beyond simple speed of locomotion measurement to further parameters requiring more in-depth analysis (Table 2). The fact that some of the measured behavioral traits were either only weakly or not at all tied to speed of locomotion (Tables 3, 4) demonstrates the potential utility of expanded data analyses in advanced analysis of *C. elegans* behavior. In turn, increased behavioral analyses allow for the quantification of behavioral change that may not sufficiently be represented by speed of locomotion. This can be used in combination with knockout *C. elegans* to increase sensitivity in detecting subtle phenotypic changes resulting from genotypic changes. In turn, this could then help in furthering the connection in *C. elegans* between genetics and behavior.

### Nested analysis

Due to the design of the experiment requiring the assaying of multiple worms per NGM-agar plate, nested analysis was used to investigate the potential impact of inter-plate variability on statistical analysis, an issue recently reviewed in the literature (Aarts et al., 2014). As summarized in Table 2, the nested factor played a statistically significant role in the observed difference between variables in nearly half (13/28) of the analyses. It has previously been suggested that, due to the high interindividual variability observed in *C. elegans* behavioral assays, a large sample number is required (Angstman et al., 2015). The present study shows that, when using a nested design to accomplish such a sample number, such a design must be accounted for due to inter-plate variability (see also Figure 2). Turned around, if this factor is now accounted for, an even greater number of *C. elegans* worms must be assayed to achieve the proper statistical power.

### Relevance in toxicology, drug discovery, and RNAi screening

The methods used in the present study also offer particular relevance in the fields of toxicology, drug discovery, and RNAi screening. Because the effects of various chemical compounds may not be predictable, the ability to screen using multiple parameters offers increased sensitivity in terms of identifying subtle behavioral change. Furthermore, potential changes in behavioral traits in *C. elegans* may be better defined and measured on agar plates rather than on worms swimming in liquid. Although novel worm tracking software for tracking *C. elegans* swimming in liquid have recently been published (Hardaway et al., 2014; Restif et al., 2014), most assays involving chemical exposure and the measurement of behavior were performed via chemicals mixed in with the agar. This is, however, not the ideal delivery method for *C. elegans* as it may require substantially increased concentrations to achieve an effect. This is due to the fact that *C. elegans* have a cuticle barrier that can result in internal concentrations on the order of magnitudes lower than external concentrations (Rand and Johnson, 1995; Davies et al., 2003; Davies and McIntire, 2004). Exposure to chemicals in liquid, however, provides a much more effective delivery method. This may also apply to RNAi screening, as the ability to carry out RNAi in *C. elegans* via soaking is less labor intensive than injection and less variable than via agar plate feeding (for details see, e.g., Ahringer, 2006).

In the present study, worms were maintained solely in a liquid culture until immediately before video capture. Importantly, we have previously demonstrated that *C. elegans* grown in liquid cultures do not demonstrate statistically significant difference in average speed of locomotion compared to those raised on NGM-agar plates (Angstman et al., 2015). Due to our rapid transfer method, previously described in Angstman et al. (2015), both advantageous scenarios can be achieved: chemical exposure in liquid and behavioral analysis on NGM-agar plates. The combination of this and enhanced capability of behavioral analysis provides an ideal model for use in the fields of toxicology, drug discovery, and RNAi screening.

In summary, the advanced analysis model of *C. elegans* behavior presented in the present study demonstrates the potential utility and effectiveness of advanced behavioral analyses. Such analyses go beyond simple speed of locomotion measurements, offering greater sensitivity in measuring *C. elegans* behavioral change. This model potentially offers utility in the connecting of *C. elegans* genetics with behavior as well as in the fields of toxicology, drug discovery, and RNAi screening, to mention only a few.

## Author contributions

NA, HF, and CS conceived and designed the experiments, NA performed experiments, and NA, HF, and CS analyzed data and wrote the manuscript.

### Conflict of interest statement

CS serves as paid consultant for MBF Bioscience (Williston, VT, USA), the manufacturer of the WormLab software that was used in the present study to analyze data. However, CS has not received financial support directly or indirectly related to this manuscript. The authors declare that the research was conducted in the absence of any commercial or financial relationships that could be construed as a potential conflict of interest.

## Supplementary material

Video S1 **Wild type (N2) *C. elegans* on an NGM-agar plate with an *E. coli* lawn**.

Video S2 **Wild type (N2) *C. elegans* on an NGM-agar plate with no lawn**.

Table S1 **Tracking settings used in WormLab software**.
