# Supplementary material for: Advanced Behavioral Analyses Show that the Presence of Food Causes Subtle Changes in C. elegans Movement
Source: Front Behav Neurosci. 2016 Mar 31;10:60. doi: 10.3389/fnbeh.2016.00060 (PMC4814519; doi:10.3389/fnbeh.2016.00060)
Supplement: Table S1 — Tracking settings used in WormLab software. [file Table1.docx]

**Table S1 | Tracking Settings Used in WormLab software (MBF Bioscience).**

| **Category** | **Setting** | **Value** |
| --- | --- | --- |
| Sequence | Captured frame rate | 15 FPS |
| info | Scale | 8.37 µm/pixel |
| Image | Threshold level^a^ | *Individually selected* |
| parameters | Image mode | Dark worms on a light background |
|  | Hotspot illumination correction  Background smoothing | Off  Off |
|  | Gaussian smoothing | Off |
|  | Fill holes  Small object filter | Off  Off |
| Detection | Detect worms at the edge of the image | Off |
| parameters | Area^b^ | *Automatically set* |
|  | Length^b^ | *Automatically set* |
|  | Width^b^ | *Automatically set* |
|  | Width/length ratio^b^ | *Automatically set* |
|  | Detection frequency | 1 |
|  | Length fitting | Off |
|  | Width fitting | On |
|  | Use Whole Plate Mode | Off |
|  | Fitting iterations | 80 |
|  | Spinal axis sample | 59 |
|  | Enforce width uniformity | Off |
| Tracking | Start frame | 1 |
| parameters | End frame | 900 |
|  | Only track currently detected worms  Use back tracking | Off  On |
|  | Track worms at the edge of the image | Off |
|  | Max tracked hypotheses  Tracking Mode | 1  Crawling |
|  | Frames worms can touch boundary | 5 |
|  | Frames worms can overlap | 50 |
|  | Position tolerance | 0.30 |
|  | Shape tolerance | 0.25 |
|  | Minimum track duration | Off |

^a^ Chosen to optimize representation of worms while limiting appearance of background

^b^ Automatically calculated based on manual identification of multiple worms
